# An efficacy comparison of anti-vascular growth factor agents and laser photocoagulation in diabetic macular edema: a network meta-analysis incorporating individual patient-level data

**DOI:** 10.1186/s12886-018-1006-9

**Published:** 2018-12-27

**Authors:** Dominic Muston, Jean-Francois Korobelnik, Tim Reason, Neil Hawkins, Ismini Chatzitheofilou, Fay Ryan, Peter K. Kaiser

**Affiliations:** 1 Bayer US LLC, Whippany, NJ USA; 2 Service d’ophtalmologie CHU, Bordeaux, France; 3 University of Bordeaux, Inserm, Bordeaux Population Health Research Center, team LEHA, Bordeaux, France; 4 QuintilesIMS, London, UK; 5 University of Glasgow, Glasgow, UK; 6 Cole Eye Institute, Cleveland, OH USA

**Keywords:** Intravitreal aflibercept, Diabetic macular edema, Intravitreal ranibizumab, Meta-analysis

## Abstract

**Background:**

This was an updated network meta-analysis (NMA) of anti-vascular endothelial growth factor (VEGF) agents and laser photocoagulation in patients with diabetic macular edema (DME). Unlike previous NMA that used meta-regression to account for potential confounding by systematic variation in treatment effect modifiers across studies, this update incorporated individual patient-level data (IPD) regression to provide more robust adjustment.

**Methods:**

An updated review was conducted to identify randomised controlled trials for inclusion in a Bayesian NMA. The network included intravitreal aflibercept (IVT-AFL) 2 mg bimonthly (2q8) after 5 initial doses, ranibizumab 0.5 mg as-needed (PRN), ranibizumab 0.5 mg treat-and-extend (T&E), and laser photocoagulation. Outcomes included in the analysis were change in best-corrected visual acuity (BCVA), measured using an Early Treatment Diabetic Retinopathy Study (ETDRS) chart, and patients with ≥10 and ≥ 15 ETDRS letter gains/losses at 12 months. Analyses were performed using networks restricted to IPD-only and IPD and aggregate data with (i) no covariable adjustment, (ii) covariable adjustment for baseline BVCA assuming common interaction effects (against reference treatment), and (iii) covariable adjustments specific to each treatment comparison (restricted to IPD-only network).

**Results:**

Thirteen trials were included in the analysis. IVT-AFL 2q8 was superior to laser in all analyses. IVT-AFL 2q8 showed strong evidence of superiority (95% credible interval [CrI] did not cross null) versus ranibizumab 0.5 mg PRN for mean change in BCVA (mean difference 5.20, 95% CrI 1.90–8.52 ETDRS letters), ≥15 ETDRS letter gain (odds ratio [OR] 2.30, 95% CrI 1.12–4.20), and ≥10 ETDRS letter loss (OR 0.25, 95% CrI 0.05–0.74) (IPD and aggregate random-effects model with baseline BCVA adjustment). IVT-AFL 2q8 was not superior to ranibizumab 0.5 mg T&E for mean change in BCVA (mean difference 5.15, 95% CrI -0.26–10.61 ETDRS letters) (IPD and aggregate random-effects model).

**Conclusions:**

This NMA, which incorporated IPD to improve analytic robustness, showed evidence of superiority of IVT-AFL 2q8 to laser and ranibizumab 0.5 mg PRN. These results were irrespective of adjustment for baseline BCVA.

**Electronic supplementary material:**

The online version of this article (10.1186/s12886-018-1006-9) contains supplementary material, which is available to authorized users.

## Background

Diabetic macular edema (DME) is the leading cause of vision loss in patients with diabetic retinopathy [1]. Treatments include anti-vascular endothelial growth factor (VEGF) agents, laser, steroids, and surgery. Anti-VEGF agents are currently standard of care in DME treatment. These agents are known to target underlying abnormalities in the VEGF signalling cascade, which is a primary pathway in DME progression [2]. Anti-VEGF agents reduce the incidence of legal blindness, and their long-term efficacy and safety have been proven in numerous randomised trials [3–8].

Direct randomised comparisons of anti-VEGF agents in DME, however, are limited to the Diabetic Retinopathy Clinical Research Network (DRCR.net) Protocol T trial [4, 8], which only included ranibizumab at 0.3 mg (US dose) and did not include ranibizumab at 0.5 mg (EU dose) and is therefore not directly relevant to retina practice outside the United States. Many comparator DME trials have also used mean change in best-corrected visual acuity (BCVA; Early Treatment Diabetic Retinopathy Study [ETDRS] letters) after 12 months as a primary efficacy measure, but differences in baseline BCVA between trials, a likely treatment effect modifier [4, 8], may confound indirect comparisons.

Previous network meta-analyses (NMA) of anti-VEGF agents [9–11] have attempted to adjust for baseline BCVA using aggregate data. These analyses provide useful data for decision-making, but meta-regression based on aggregate data may not mirror effects at the patient level and could also be confounded by non-linear covariable effects and other sources of heterogeneity between trials that are not accounted for by the variables included in the meta-regression [12, 13]. This bias is known as ecological bias.

The European Medicines Agency has recently issued a policy with a primary objective of making clinical reports and individual patient-level data (IPD) available to enable public scrutiny and allow for the application of new knowledge in future research [14]. Within-trial regression analysis based on IPD provides more robust adjustment for differences in BCVA between trials. However, to our knowledge, no published NMA has incorporated IPD.

The objective of this NMA was to perform an updated indirect comparison of the efficacy of approved anti-VEGF regimens and laser photocoagulation in DME based on mean change in BCVA and gain or loss of letters at 12 months. The NMA incorporated both aggregate data and IPD, where available. Different regimens, including fixed, as-needed (PRN), and treat-and-extend (T&E), were also included. These regimens have not been incorporated in detail in previous NMAs.

## Methods

### Search strategy

**Fig. 1:**
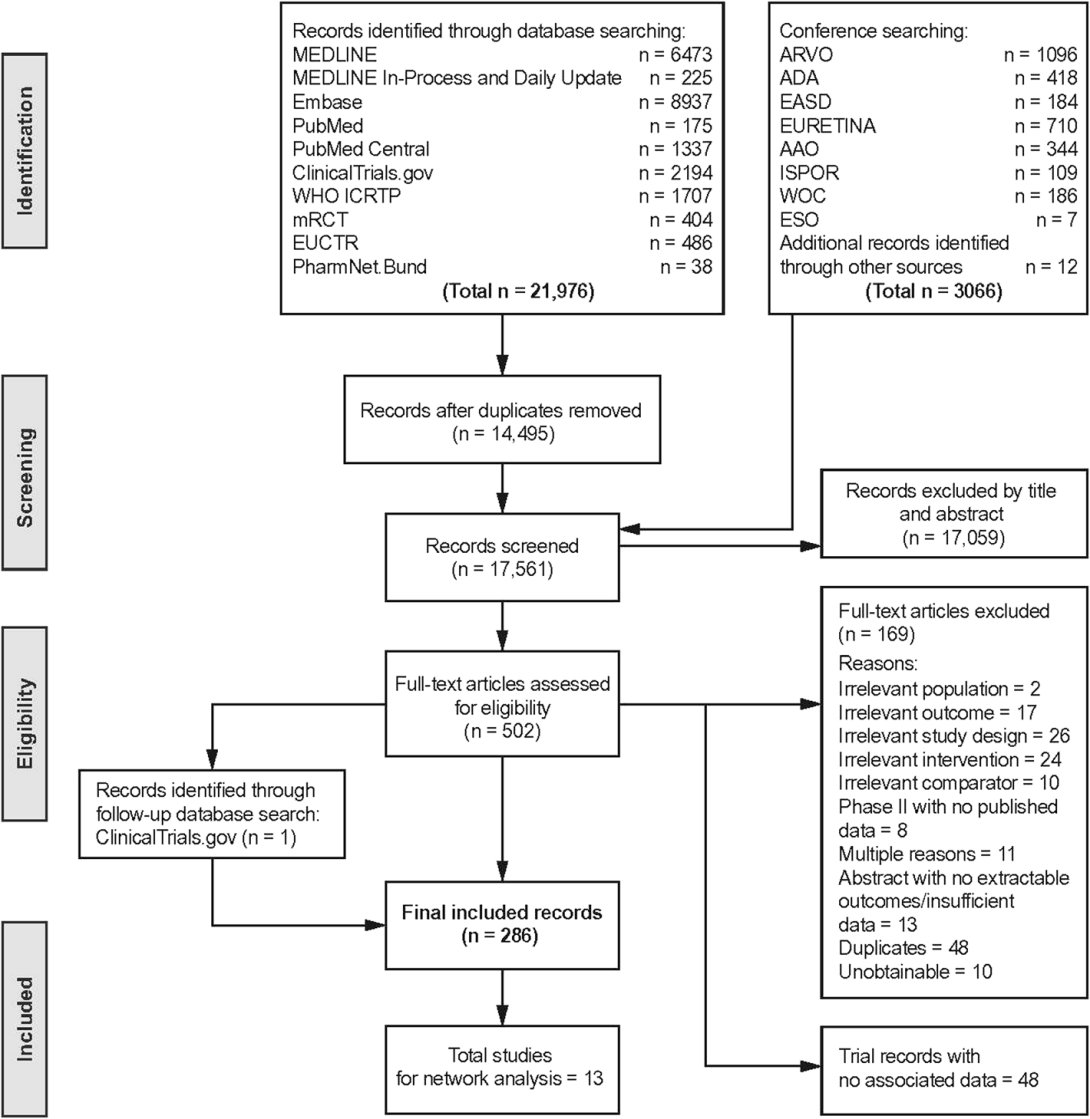
Flow chart summarizing the literature search

The literature search was updated from a previous systematic literature review performed in February 2015 [9]. This updated literature review identified all relevant randomised controlled trials (RCTs) that were published from February 2015 to December 2016 using the same search strategy that was described in the previous publication [9]. An additional search of ClinicalTrials.gov (from January 2015 to December 2016) was also performed to identify any new trials. This was performed by searching for ‘diabetic macular edema phase III RCT’ to be consistent with the previous analysis. As summarized in Fig. 1, the analysis presented here uses data from all studies identified above.

### Inclusion criteria

Trials were screened using the populations, interventions, comparators, outcomes, and study design (PICOS) criteria as defined in the previous publication [9]. This approach also applied to the results from the ClinicalTrials.gov search. Only trials that reported the randomised controlled results at 12 months for mean change from baseline in BCVA (ETDRS letters) (continuous outcome), and the proportion of patients achieving gain/loss of ≥10 and ≥ 15 ETDRS letters (binary outcomes) were included.

Treatment regimens of interest for European practice were IVT-AFL 2 mg every 8 weeks (2q8) after 5 initial doses, ranibizumab 0.5 mg PRN, ranibizumab 0.5 mg T&E, and laser photocoagulation. Because it is not licensed for ophthalmic use, bevacizumab was not included in the analysis. Treatments were grouped according to dose, number of loading doses, regimen, which could be proactive (fixed), reactive (PRN), or proactive/reactive (T&E), and median number of injections. Treatment regimens for ranibizumab and IVT-AFL were not the same because treatment regimens were determined by the individual study sponsors. These groupings were used for treatment classification in the NMA. All included trials were compliant with the Declaration of Helsinki, had protocols approved by relevant country- and trial-specific institutional review boards/independent ethics committees, and enrolled patients who provided written informed consent for participation.

### Data extraction

Data extraction was based on methods described previously [9]. Missing standard errors (SEs) for the change in continuous outcome were imputed using the following equation [15]:

1 $$ {SE}_{change}=\frac{\sqrt{SD_{baseline}^2+{SD}_{final}^2-\left(2\times \mathrm{correlation}\times {SD}_{baseline}\times {SD}_{final}\right)\ }}{\sqrt{sample\ size}} $$

Where *SD*_*final*_ was not available, *SD*_*baseline*_ was used in place of *SD*_*final*_. For the correlation, 0.5 was used in the formula above; this has been described as a conservative assumption [16]. Standard deviation (SD) and 95% confidence intervals (CIs) were converted to SEs based on established formulas ([15]. If CI, SD, and SE were not reported and only *P* values were available, it was assumed that the *P* values were calculated using a Wald test. The SE was then calculated by rearranging the formula for calculating *P* values for normally distributed variables.

### Analyses

The trials were used to form a Bayesian NMA for continuous and binary outcomes. Analyses were performed using IPD-only and IPD and aggregate data networks with (i) no covariable adjustment, (ii) covariable adjustment for baseline BVCA assuming common interaction effects (against reference treatment), and (iii) covariable adjustments specific to each treatment comparison (IPD-only). Both fixed-effect and random-effects models were fitted.

Baseline BCVA was identified as the main treatment effect modifier (covariable) for vision-related outcomes, and was controlled for in the NMA. Based on previous analysis of IPD from the VIVID-DME and VISTA-DME trials, other potential covariables (diabetic retinopathy severity scale, prior anti-VEGF treatment, baseline glycated haemoglobin, blood pressure treatment, and cataracts) did not have a significant impact on IVT-AFL treatment effects and were not included [17–21]. Baseline central retinal thickness (CRT) was shown to be a potential treatment effect modifier on vision-related outcomes when receiving IVT-AFL treatment [22]; however, it was excluded as it was found to be highly correlated with baseline BCVA, which would have resulted in co-linearity in the model. An additional file contains further information on the models and covariable adjustment (see Additional file 1: Appendix 1).

The analyses were conducted with OpenBUGS version 3.2.3 using Monte Carlo chain simulations to calculate posterior distributions for the parameters of interest. A Monte Carlo error of less than 5% of the posterior SD indicated acceptable simulation error [23]. The absolute and relative model fits were assessed using residual deviance and deviance information criterion statistics. Convergence was assessed by visual inspection of caterpillar and probability density plots and by running analyses using 3 separate chains to ensure that all chains converged to the same distribution. We ran all models with a burn-in of 50,000 and 200,000 iterations.

All baseline and intervention parameters were given vague normal (mean = 0, SD = 1000) distributions, an appropriately large range given the scale of measurement. A binomial likelihood with logit link function was used for binary data, and a normal likelihood with identity link function was used for continuous data. The OpenBUGS codes used were adapted from previously published codes [24]. This allowed the code to be easily adapted to include IPD. The methodology also followed guidance from the International Society for Pharmacoeconomics and Outcomes Research Task Force on Indirect Treatment Comparisons [25, 26].

Binary outcomes were reported as odds ratios (ORs), and the continuous outcome was reported as the mean treatment difference (ETDRS letters). Uncertainty was reported using 95% credible intervals (CrIs). IVT-AFL 2q8 was chosen as the reference treatment so that it could be compared with all treatments of interest (ie, ranibizumab 0.5 mg PRN, ranibizumab 0.5 mg T&E, and laser photocoagulation).

## Results

### Networks

**Fig. 2:**
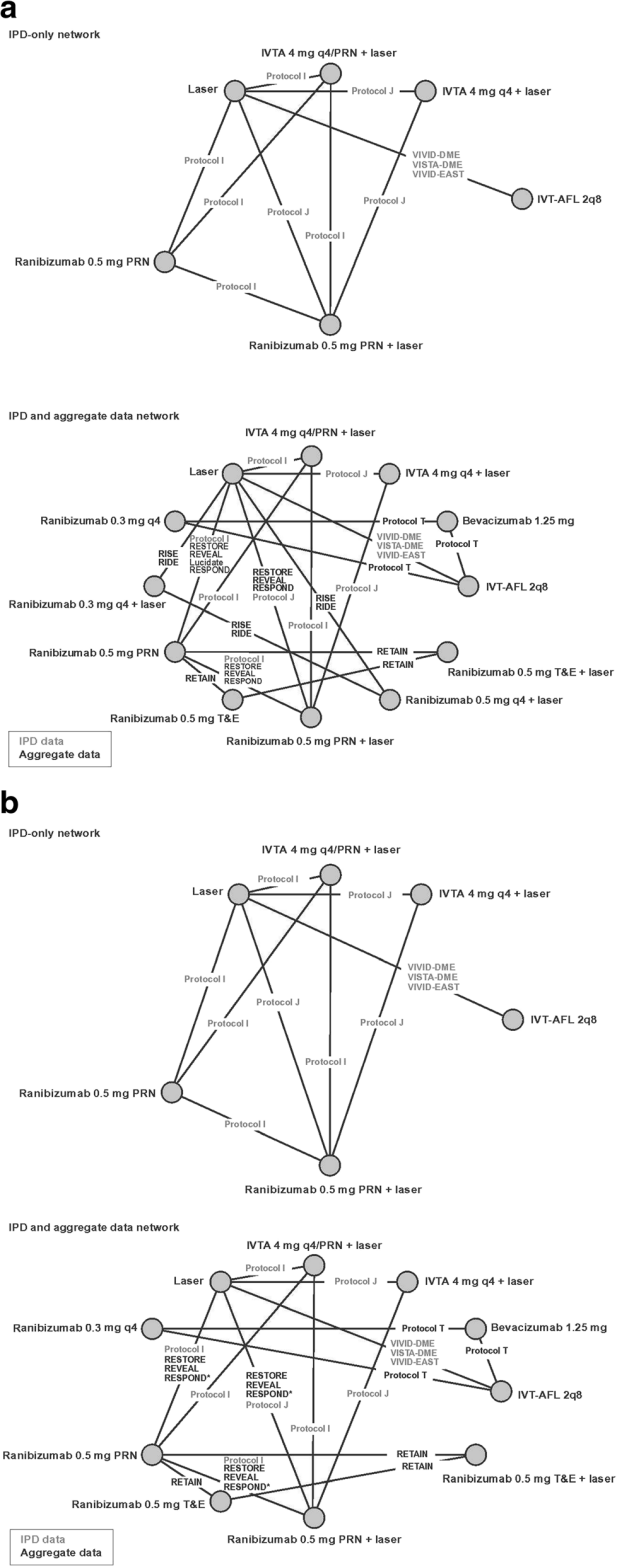
IPD-only and IPD and aggregate data networks for (**a**) mean change in BCVA (continuous outcome) and (**b**) gain/loss of ≥10 and ≥ 15 ETDRS letters (binary outcomes). 2q8, every 8 weeks; BCVA, best-corrected visual acuity; ETDRS, Early Treatment Diabetic Retinopathy Study; IPD, individual patient-level data; IVTA, intravitreal triamcinolone acetonide; IVT-AFL, intravitreal aflibercept; PRN, as-needed; q4, every 4 weeks; T&E, treat-and-extend. ^a^RESPOND did not provide data for letter losses. Note: outcomes for ranibizumab 0.3 mg (US dose), intravitreal triamcinolone acetonide and bevacizumab are not reported

A total of 13 trials were identified as eligible for inclusion [4–7, 27–33]. The treatment regimens in these trials were classified and included in the NMA as summarised in Additional file 2: Appendix 2; an overview of the outcomes reported in these trials is summarised in Additional file 3: Appendix 3. Thirteen trials provided continuous outcome data (ie, mean change in BCVA) and 10 trials provided binary outcome data (ie, proportion of patients with loss/gain of ≥10 and/or ≥ 15 ETDRS letters) as summarised in Table 1. Baseline BCVA was adjusted for by incorporating IPD from 5 trials and aggregate data from 8 trials. IPD-only and IPD and aggregate data networks were developed using these data, which are summarised in Fig. 2.

**Table 1:** Overview of trial data included in each analysis [4–8, 27–32]

IPD and aggregate network	IPD-only network
DRCR.net Protocol I	DRCR.net Protocol I
DRCR.net Protocol J	DRCR.net Protocol J
DRCR.net Protocol T	VISTA-DME
LUCIDATE^a^	VIVID-DME
RESPOND^b^	VIVID-EAST
RESTORE	
RETAIN	
REVEAL	
RIDE^a^	
RISE^a^	
VISTA-DME	
VIVID-DME	
VIVID-EAST	

^a^LUCIDATE, RISE and RIDE did not provide data for letter gains/losses

^b^RESPOND did not provide data for letter losses

In all analyses, IVT-AFL 2q8 was shown to be superior to laser. Overall, the means for the coefficients describing the interactions effects were negative (see Additional file 1: Appendix 1) indicating that patients with lower BCVA at baseline had a greater response to treatment. Only random-effects IPD and aggregate models are described here.

### Mean change in BCVA (continuous outcome)

The results for the mean change in BCVA (ETDRS letters) from the analysis of the IPD-only and IPD and aggregate data networks are shown in Table 2. IVT-AFL 2q8 showed strong evidence of superiority (95% CrI did not cross 0) versus laser, ranibizumab 0.5 mg PRN, and ranibizumab 0.5 mg PRN with laser in the IPD and aggregate networks (all covariable adjustments) for mean change in BCVA. The mean difference (95% CrI) in BCVA (ETDRS letters) for IVT-AFL 2q8 versus ranibizumab 0.5 mg PRN was 5.15 (1.82–8.54) (no covariable adjustment) and 5.20 (1.90–8.52) (common covariable adjustment). IVT-AFL 2q8 was not superior to ranibizumab 0.5 mg T&E for mean change in BCVA (mean difference 5.15; 95% CrI -0.26–10.61 ETDRS letters) (IPD and aggregate random-effects model).

**Table 2:** IPD-only and IPD and aggregate network results for mean change in BCVA (ETDRS letters)

			IPD-only network		IPD and aggregate network	
Comparison	Covariable adjustment	Model	Mean difference	95% Crl	Mean difference	95% Crl
IVT-AFL 2q8 vs laser	None	Fixed	+ 10.32^a^	8.31–12.34	+ 10.32^a^	8.30–12.35
		Random	+ 10.47^a^	6.92–14.16	+ 10.45^a^	7.81–13.12
	Common (baseline BCVA)	Fixed	+ 10.30^a^	8.82–11.78	+ 10.27^a^	8.79–11.74
		Random	+ 10.54^a^	7.07–14.42	+ 10.47^a^	7.88–13.11
	Treatment-specific	Fixed	+ 10.48^a^	8.99–11.97	–	–
		Random	+ 10.65^a^	7.13–14.34		
IVT-AFL 2q8 vs ranibizumab 0.5 mg PRN	None	Fixed	+ 3.92^a^	0.60–7.24	+ 4.42^a^	2.07–6.76
		Random	+ 3.47	−3.14–10.44	+ 5.15^a^	1.82–8.54
	Common (baseline BCVA)	Fixed	+ 3.14^a^	0.48–5.80	+ 4.95^a^	3.09–6.83
		Random	+ 3.54	−2.93–10.81	+ 5.20^a^	1.90–8.52
	Treatment-specific	Fixed	+ 3.09^a^	0.44–5.72	–	–
		Random	+ 3.38	−3.20–10.18		
IVT-AFL 2q8 vs ranibizumab 0.5 mg PRN + laser	None	Fixed	+ 5.96^a^	2.65–9.25	+ 5.17^a^	2.73–7.61
		Random	+ 6.05^a^	0.45–12.19	+ 5.95^a^	2.60–9.37
	Common (baseline BCVA)	Fixed	+ 5.71^a^	3.11–8.29	+ 5.77^a^	3.81–7.74
		Random	+ 6.16^a^	0.67–12.55	+ 6.00^a^	2.66–9.36
	Treatment-specific	Fixed	+ 5.68^a^	3.06–8.29	–	–
		Random	+ 6.03^a^	0.51–11.94		
IVT-AFL 2q8 vs ranibizumab 0.5 mg T&E	None	Fixed	–	–	+ 5.06^a^	1.88–8.23
		Random			+ 5.07	−0.37–10.54
	Common (baseline BCVA)	Fixed	–	–	+ 4.90^a^	2.06–7.72
		Random			+ 5.15	−0.26–10.61
	Treatment-specific	Fixed	–	–	–	–
		Random				
IVT-AFL 2q8 vs ranibizumab 0.5 mg T&E + laser	None	Fixed	–	–	+ 5.07^a^	2.00–8.13
		Random			+ 3.80	− 1.57–9.25
	Common (baseline BCVA)	Fixed	–	–	+ 3.65^a^	0.94–6.35
		Random			+ 3.90	−1.42–9.25
	Treatment-specific	Fixed	–	–	–	–
		Random				

^a^IVT-AFL showed statistical superiority to the comparator as 95% CrI ranges did not cross 0

### Proportion of patients gaining or losing ≥10 and ≥ 15 ETDRS letters (binary outcomes)

The IPD-only and IPD and aggregate data network results for letter gains are shown in Table 3. IVT-AFL 2q8 showed strong evidence of superiority (95% CrI did not cross 1) for the proportion of patients gaining ≥15 ETDRS letters versus laser, ranibizumab 0.5 mg PRN, ranibizumab 0.5 mg PRN with laser, and ranibizumab 0.5 mg T&E with laser in the IPD and aggregate networks (all covariable adjustments). The OR (95% CrI) for gaining ≥15 ETDRS letters for IVT-AFL 2q8 versus ranibizumab 0.5 mg PRN was 2.08 (1.06–3.70) (no covariable adjustment) and 2.30 (1.12–4.20) (common covariable adjustment).

**Table 3:** IPD-only and IPD and aggregate network results for proportion of patients gaining ≥10 and ≥ 15 ETDRS letters

			Gain ≥10 ETDRS letters		Gain ≥15 ETDRS letters	
IPD-only network Comparison	Covariable adjustment	Model	OR	95% Crl	OR	95% Crl
IVT-AFL 2q8 vs laser	None	Fixed	4.82^a^	3.52–6.46	4.97^a^	3.32–7.26
		Random	18.72^a^	1.16–20.20	5.76^a^	1.97–12.24
	Common (baseline BCVA)	Fixed	4.96^a^	3.57–6.73	5.49^a^	3.47–8.38
		Random	60.90^a^	1.06–26.91	7.58^a^	1.79–15.34
	Treatment-specific	Fixed	5.10^a^	3.67–6.94	5.64^a^	3.55–8.66
		Random	7.28^a^	1.16–24.20	534.90^a^	1.85–19.79
IVT-AFL 2q8 vs ranibizumab 0.5 mg PRN	None	Fixed	2.10^a^	1.19–3.45	2.59^a^	1.29–4.69
		Random	93,680.00	0.10–29.60	10.68	0.53–14.63
	Common (baseline BCVA)	Fixed	2.09^a^	1.15–3.48	2.85^a^	1.35–5.35
		Random	32.25	0.08–42.16	59.97	0.37–20.4
	Treatment-specific	Fixed	1.99^a^	1.07–3.38	3.06^a^	1.43–5.85
		Random	594.80	0.14–43.29	2056.00	0.43–39.48
IVT-AFL 2q8 vs ranibizumab 0.5 mg PRN + laser	None	Fixed	3.04^a^	1.82–4.77	2.74^a^	1.44–4.78
		Random	22.24	0.29–31.29	5.93	0.76–13.29
	Common (baseline BCVA)	Fixed	2.92^a^	1.70–4.68	2.88^a^	1.44–5.23
		Random	969.00	0.28–41.36	10.13	0.59–16.11
	Treatment-specific	Fixed	2.99^a^	1.73–4.81	2.99^a^	1.48–5.44
		Random	22.32	0.37–47.51	52.20	0.60–23.38
			Gain ≥10 ETDRS letters		Gain ≥15 ETDRS letters	
IPD and aggregate network Comparison	Covariable adjustment	Model	OR	95% Crl	OR	95% Crl
IVT-AFL 2q8 vs laser	None	Fixed	4.82^a^	3.52–6.45	4.97^a^	3.32–7.28
		Random	5.06^a^	2.39–9.49	5.03^a^	3.09–7.88
	Common (baseline BCVA)	Fixed	4.93^a^	3.55–6.72	5.58^a^	3.52–8.58
		Random	5.27^a^	2.44–9.91	5.62^a^	3.28–9.15
IVT-AFL 2q8 vs ranibizumab 0.5 mg PRN	None	Fixed	1.72^a^	1.11–2.53	2.07^a^	1.19–3.38
		Random	1.76	0.62–3.94	2.08^a^	1.06–3.70
	Common (baseline BCVA)	Fixed	1.71^a^	1.09–2.56	2.28^a^	1.25–3.85
		Random	1.79	0.63–4.06	2.30^a^	1.12–4.20
IVT-AFL 2q8 vs ranibizumab 0.5 mg PRN + laser	None	Fixed	2.01^a^	1.32–2.94	2.11^a^	1.22–3.42
		Random	2.18	0.81–4.78	2.14^a^	1.12–3.76
	Common (baseline BCVA)	Fixed	1.96^a^	1.26–2.92	2.27^a^	1.26–3.83
		Random	2.20	0.81–4.90	2.31^a^	1.15–4.20
IVT-AFL 2q8 vs ranibizumab 0.5 mg T&E	None	Fixed	1.57	0.76–2.86	1.78	0.76–3.55
		Random	1.88	0.30–6.26	1.86	0.63–4.18
	Common (baseline BCVA)	Fixed	1.55	0.75–2.85	1.96	0.81–4.00
		Random	1.95	0.32–6.47	2.04	0.67–4.77
IVT-AFL 2q8 vs ranibizumab 0.5 mg T&E + laser	None	Fixed	2.41^a^	1.15–4.48	3.22^a^	1.32–6.68
		Random	2.88	0.47–9.68	3.35^a^	1.09–7.84
	Common (baseline BCVA)	Fixed	2.39^a^	1.13–4.48	3.56^a^	1.42–7.53
		Random	3.02	0.48–9.87	3.73^a^	1.16–8.88

^a^IVT-AFL showed statistical superiority to the comparator as 95% CrI ranges did not cross 1

The IPD-only and IPD and aggregate data network results for the proportion of patients losing ≥10 and ≥ 15 ETDRS letters are shown in Table 4. IVT-AFL 2q8 showed strong evidence of superiority (95% Crl did not cross 1) for reducing the proportion of patients losing ≥10 or ≥ 15 ETDRS letters versus laser. IVT-AFL 2q8 was also superior to ranibizumab 0.5 mg PRN, ranibizumab 0.5 mg PRN with laser, and ranibizumab 0.5 mg T&E in the IPD and aggregate networks (all covariable adjustments) for reducing ≥10 ETDRS letter losses. The OR (95% Crl) for losing ≥10 ETDRS letters for IVT-AFL 2q8 versus ranibizumab 0.5 mg T&E was 0.19 (0.00–0.96) (no covariable adjustment) and 0.21 (0.00–0.97) (common covariable adjustment). However, a number of OR values calculated were implausible, which may be attributable to reduced evidence supporting comparison for ETDRS letters in Table 3 and Table 4. These implausible values are limited to certain analyses of the IPD-only dataset, and may be a result of the methodology employed.

**Table 4:** IPD-only and IPD and aggregate network results for patients losing ≥10 and ≥ 15 ETDRS letters

			Loss ≥10 ETDRS letters		Loss ≥15 ETDRS letters	
IPD-only network Comparison	Covariable adjustment	Model	OR	95% Crl	OR	95% Crl
IVT-AFL vs laser	None	Fixed	0.06^a^	0.02–0.12	0.04^a^	0.01–0.112
		Random	0.11^a^	0.01–0.29	0.22^a^	0.00–0.28
	Common (baseline BCVA)	Fixed	0.06^a^	0.02–0.12	0.04^a^	0.00–0.11
		Random	0.19^a^	0.01–0.27	0.07^a^	0.00–0.24
	Treatment-specific	Fixed	0.05^a^	0.02–0.11	0.02^a^	0.00–0.08
		Random	0.08^a^	0.01–0.24	0.04^a^	0.00–0.16
IVT-AFL vs ranibizumab 0.5 mg PRN	None	Fixed	0.37	0.07–1.22	0.51	0.02–2.51
		Random	92.49	0.01–8.70	278,400.00	0.00–39.31
	Common (baseline BCVA)	Fixed	0.37	0.07–1.24	0.47	0.02–2.34
		Random	483.00	0.01–6.20	825.00	0.00–22.22
	Treatment-specific	Fixed	0.51	0.07–2.07	0.45	0.00–2.59
		Random	9.28	0.01–7.53	300.00	0.00–15.68
IVT-AFL vs ranibizumab 0.5 mg PRN + laser	None	Fixed	0.17^a^	0.04–0.44	0.23^a^	0.02–0.91
		Random	6.74	0.01–2.11	74.93	0.00–7.32
	Common (baseline BCVA)	Fixed	0.18^a^	0.04–0.47	0.20^a^	0.02–0.81
		Random	12.89	0.01–1.75	11.07	0.00–5.17
	Treatment-specific	Fixed	0.18^a^	0.04–0.51	0.13^a^	0.00–0.65
		Random	3.38	0.01–1.89	5.42	0.00–3.04
			Loss ≥10 ETDRS letters		Loss ≥15 ETDRS letters	
IPD and aggregate network Comparison	Covariable adjustment	Model	OR	95% Crl	OR	95% Crl
IVT-AFL 2q8 vs laser	None	Fixed	0.06^a^	0.02–0.12	0.04^a^	0.00–0.12
		Random	0.06^a^	0.02–0.14	0.04^a^	0.00–0.14
	Common (baseline BCVA)	Fixed	0.06^a^	0.02–0.12	0.04^a^	0.00–0.11
		Random	0.06^a^	0.02–0.14	0.04^a^	0.00–0.13
IVT-AFL 2q8 vs ranibizumab 0.5 mg PRN	None	Fixed	0.24^a^	0.07–0.60	0.31	0.02–1.15
		Random	0.25^a^	0.05–0.73	0.36	0.02–1.49
	Common (baseline BCVA)	Fixed	0.24^a^	0.07–0.59	0.28	0.02–1.05
		Random	0.25^a^	0.05–0.74	0.33	0.02–1.39
IVT-AFL 2q8 vs ranibizumab 0.5 mg PRN + laser	None	Fixed	0.14^a^	0.04–0.33	0.11^a^	0.01–0.37
		Random	0.15^a^	0.03–0.41	0.13^a^	0.01–0.47
	Common (baseline BCVA)	Fixed	0.14^a^	0.04–0.33	0.10^a^	0.01–0.34
		Random	0.15^a^	0.03–0.41	0.11^a^	0.01–0.43
IVT-AFL 2q8 vs ranibizumab 0.5 mg T&E	None	Fixed	0.15^a^	0.01–0.75	0.33	0.00–1.94
		Random	0.19^a^	0.00–0.96	0.81	0.00–3.00
	Common (baseline BCVA)	Fixed	0.15^a^	0.00–0.73	0.30	0.00–1.74
		Random	0.21^a^	0.00–0.97	0.54	0.00–2.67
IVT-AFL 2q8 vs ranibizumab 0.5 mg T&E + laser	None	Fixed	222,500.00	0.03–607.2	201,200.00	0.02–671.3
		Random	325,000.00	0.02–589.9	130,000.00	0.01–751.0
	Common (baseline BCVA)	Fixed	470,400.00	0.03–555.1	3,311,000.00	0.02–592.0
		Random	662,700.00	0.02–622.8	2,877,000.00	0.01–775.5

^a^IVT-AFL showed statistical superiority to the comparator as 95% CrI ranges did not cross 1

## Discussion

It is important for physicians and policymakers to compare the relative efficacy of DME treatments using the most robust methods available. Although direct head-to-head comparative trials provide stronger internal validity than indirect comparisons, there are a small number of these trials in DME (eg, DRCR.net Protocol T) [4, 8], and there are no direct randomised comparative trial outcomes of IVT-AFL 2q8 and ranibizumab 0.5 mg (any regimen) available. The objective of this NMA was to perform an updated indirect comparison of IVT-AFL 2q8 versus relevant comparators, including ranibizumab 0.5 mg, from a previous publication [9]. Notably, this NMA incorporated IPD where available and adjusted for baseline BCVA, which is a known and important treatment effect modifier. To our knowledge, this is also the first NMA in DME that adjusted for any imbalance of treatment effect modifiers using IPD. Previous analyses that used only aggregate data are prone to ecological bias.

In the IPD and aggregate random-effects models, IVT-AFL 2q8 (after 5 monthly loading doses) showed strong evidence of superiority versus laser and ranibizumab 0.5 mg PRN with/without laser for mean change in BCVA from baseline and for gain of ≥15 ETDRS letters. IVT-AFL 2q8 also showed strong evidence of superiority for reducing ≥10 ETDRS letter losses versus ranibizumab 0.5 mg PRN with/without laser and versus ranibizumab 0.5 mg T&E. These results were consistent regardless of whether adjustment was made for baseline BCVA.

The BCVA results are similar to those in the previously published NMA that included fewer trials and did not include IPD [9]. In this previous NMA, there was an increase in mean BCVA at 12 months with IVT-AFL 2q8 over ranibizumab 0.5 mg PRN of 4.67 letters (95% CrI 1.85–7.52) in the random-effects Bayesian model (10 trials). However, there was no significant difference between IVT-AFL 2q8 versus ranibizumab 0.5 mg PRN for gain of ≥15 ETDRS letters (OR 1.87, 95% Crl 0.87–4.16) or loss of ≥10 ETDRS letters (OR 0.26, 95% Crl 0.05–1.31) in the random-effects Bayesian model (6 trials). The BCVA results are also consistent with those in a recent NMA that used an aggregate data approach [34]. This NMA of 21 trials (4307 eyes), which compared 11 different DME interventions including IVT-AFL and ranibizumab 0.5 mg, showed that IVT-AFL was the most favourable treatment at 12 months with respect to improvements in BCVA (OR 8.19, 95% CrI 5.07–11.96) and central macular thickness (OR -110.83, 95% CrI -190.25 to − 35.27) [34].

The availability and use of IPD can be important for ensuring that NMAs provide more robust adjustment for differences in between-trial baseline BCVA. In our NMA, we showed that adjustment for baseline BCVA did not affect the results in any model, but a lower BCVA at baseline was associated with a greater response to treatment. These findings are comparable with those observed in DRCR.net Protocol T [4]. In DRCR.net Protocol T, the mean change in visual acuity score (VAS) at 12 months was greater in patients with a lower BCVA score at baseline; for patients with baseline VAS of 74–78, the mean change in VAS was ~ 7 letters (IVT-AFL 2 mg) and ~ 6.5 letters (ranibizumab 0.3 mg) at 12 months, and for patients with baseline VAS of 24–53 letters the mean change in VAS was ~ 25 letters (IVT-AFL 2 mg) and ~ 17.5 letters (ranibizumab 0.3 mg) [4]. Similar findings were observed in a post hoc analysis of the VISTA-DME and VIVID-DME trials, IVT-AFL 2q8 treatment resulted in slightly better 12-month visual acuity outcomes in patients with baseline BCVA of ≥39 to ≤60 letters compared with those with a baseline BCVA of ≥61 to ≤73 letters; the mean change with IVT-AFL 2q8 at 12 months was 11.3 ETDRS letters (VIVID-DME) and 11.4 ETDRS letters (VISTA-DME) (baseline BCVA of ≥39 to ≤60 letters) and 8.6 ETDRS letters (VIVID-DME) and 9.5 ETDRS letters (VISTA-DME) (baseline BCVA of ≥61 to ≤73 letters) [35].

The comparison of IVT-AFL 2q8 versus ranibizumab 0.5 mg (PRN and T&E) also provides a useful complement to the direct comparative evidence of IVT-AFL 2 mg versus ranibizumab 0.3 mg from DRCR.net Protocol T [4, 8]. The EURETINA guidelines question the extent to which the slower effect of ranibizumab seen in DRCR.net Protocol T compared with IVT-AFL is attributable to the lower dose used [36]. This outcome is consistent with those in other published NMAs [9, 37]. In an updated Cochrane analysis of 24 trials (6007 patients with DME) [37], DME patients receiving ranibizumab (0.3 mg and 0.5 mg monthly) were less likely to gain ≥3 lines of visual acuity at 1 year compared with IVT-AFL (risk ratio [RR] 0.75, 95% CI 0.60–0.94). At 1 year, the visual acuity and CRT were worse with ranibizumab compared with IVT-AFL (mean difference in visual acuity: 0.08 logMAR, 95% CI 0.05–0.11; mean difference in CRT: 39 μm, 95% CI 2–76). It should be noted that ranibizumab 0.3 mg and 0.5 mg monthly were merged into 1 group as no heterogeneity was suspected between studies using these regimens in this Cochrane analysis. These authors concluded that the difference between IVT-AFL and ranibizumab was consistent with indirect evidence using ranibizumab 0.5 mg.

Despite the robust approach used in the current analysis, there are still some limitations associated with this updated NMA. We did not consider safety outcomes or longer-term efficacy outcomes, and there was limited availability of IPD in the public domain. Outcomes could also have been improved with the replacement of aggregate data from some trials (such as DRCR.net Protocol T) with IPD; however, the availability of IPD from DRCR.net Protocols I and J in addition to VIVID-DME, VISTA-DME, and VIVID-EAST enabled sufficient IPD to be included.

## Conclusions

This NMA, which incorporated aggregate data and IPD to improve model robustness, consistently showed evidence of superiority of IVT-AFL 2q8 to laser and ranibizumab 0.5 mg PRN with/without laser for mean change in BCVA, gain of ≥15 ETDRS letters, and loss of ≥10 ETDRS letters at 12 months. IVT-AFL 2q8 was also superior to ranibizumab 0.5 mg T&E for loss of ≥10 ETDRS letters. These efficacy results were consistent irrespective of adjustment for baseline BCVA. Comparison of IVT-AFL 2q8 versus ranibizumab 0.5 mg PRN and T&E provides a useful complement to the direct comparative evidence of IVT-AFL 2 mg versus ranibizumab 0.3 mg given in DRCR.net Protocol T. It is hoped that these data will be of additional benefit to those involved in the care of DME patients and to policymakers interested in developing future NMAs.

## Additional files

Additional file 1: **Appendix 1.** Statistical methods and covariable adjustments. (DOCX 57 kb)

Additional file 2: **Appendix 2.** Treatment regimens classified and included in the NMA. (DOCX 64 kb)

Additional file 3: **Appendix 3.** Overview of outcomes of studies included in the NMA. (DOCX 68 kb)

## Abbreviations

2q8: 2 mg bimonthly
BCVA: Best-corrected visual acuity
CI: Confidence interval
CrI: Credible interval
CRT: Central retinal thickness
DME: Diabetic macular edema
DRCR.net: Diabetic Retinopathy Clinical Research Network
ETDRS: Early Treatment Diabetic Retinopathy Study
IPD: Individual patient-level data
IVT-AFL: Intravitreal aflibercept
NMA: Network meta-analysis
OR: Odds ratio
PICOS: Populations, interventions, comparators, outcomes and study design
PRN: As-needed
RCT: Randomised controlled trial
SD: Standard deviation
SE: Standard error
T&E: Treat-and-extend
VAS: Visual acuity score
VEGF: Vascular endothelial growth factor
