# Supplementary material for: An efficacy comparison of anti-vascular growth factor agents and laser photocoagulation in diabetic macular edema: a network meta-analysis incorporating individual patient-level data
Source: BMC Ophthalmol. 2018 Dec 27;18:340. doi: 10.1186/s12886-018-1006-9 (PMC6307247; doi:10.1186/s12886-018-1006-9)
Supplement: Supplementary file 1 — Appendix 1. Statistical methods and covariable adjustments. (DOCX 57 kb) [file 12886_2018_1006_MOESM1_ESM.docx]

Additional file 1

Appendix 1

Statistical methods and covariable adjustment

This supplement describes further the statistical methods adopted in this work and provides additional information on the results relating to the covariable adjustment.

# General framework

First, we aim to provide a general framework for the statistical models explored, beginning with notation and data structure for binary and continuous outcomes. This follows Hawkins et al, 2016 [1].

## Notation

We use the following notation to describe the network meta-analysis (NMA) parameterisations: individual patients or study treatment arms are denoted by, where *I* is the total number of arms or individual patients in the studies in the network. $s[i]$ and $t[i]$ denote the study and treatment associated with the individual patient or study arm *i*. This notation is used to avoid double subscripting. The treatment effect for treatment *t* compared with the reference treatment (*t=1*) is denoted by the parameter$d_{t}$, with $d_{1}=0$ for identification.

Individual patients or study treatment arms may have an associated vector of covariables, $\underline{x[i]}$. In this analysis, only one treatment-effect covariable was included (baseline best-corrected visual acuity [BCVA]), so the covariable is a scalar, $x[i]$.

## Binary outcome data

Binary data are represented by $r\left[ i \right]$ and $n\left[ i \right]$. For individual patients, $r\left[ i \right]$ equals 0 or 1 depending on whether the patient is a “responder” or not; and $n\left[ i \right]$ equals 1. For study treatment arms, $r\left[ i \right]$ and $n\left[ i \right]$ represent the total number of responders and patients, respectively.

A binomial distribution is assumed for binary data:

$r\left[ i \right]\sim Bin(p\left[ i \right],n\left[ i \right])$ [1]

where $p[i]$ is the probability of an event in study $s[i]$ under treatment$t[i]$. The probability $p[i]$ is derived from the predicted log-odds of an event $n\left[ i \right]$:

$p\left[ i \right]=\frac{exp\left( \eta\left[ i \right] \right)}{1+exp\left( \eta\left[ i \right] \right)}$ [2]

## Continuous outcome data

Continuous data are represented in the following manner. For individual patients, $y[i]$ is the observed response for the individual and $z_{s\left[ i \right], t[i]}^{2}$ is the estimated standard deviation for the study and treatment arm relevant to patient *i*. For study treatment arms, $y[i]$ is the mean response for the treatment arm *i* and $z_{s\left[ i \right], t[i]}^{2}$ is the reported standard error for the study and treatment arm individual patient data or the reported standard error for study arm data.

A normal distribution is assumed for continuous data:

$y\left[ i \right]\sim Norm(\eta\left[ i \right], z_{s\left[ i \right], t\left[ i \right]}^{2})$ [3]

where $\eta\left[ i \right]$ is the predicted mean response.

## Fixed treatment effects model

The predicted log odds of an event or the mean response, $\eta_{i}$, are estimated as the sum of study and treatment specific effects:

$\eta\left[ i \right]=\mu_{s\left[ i \right]}+d_{t\left[ i \right]}+\alpha.x\left[ i \right]+\beta_{t\left[ i \right]}.x\left[ i \right]$ [4]

The variation in $\mu_{s[i]}$ across studies provides a measure of the heterogeneity in reference treatment risk across studies and can provide some indication of whether heterogeneity and/or inconsistency are likely to be present [2]. As in contrast-based parameterisation, this term is included to ensure that between-study differences in absolute response do not affect estimates of treatment effect.

The regression analysis includes both main effect terms $\alpha_{s[i]}$ describing the direct effect of covariables on treatment and interaction terms $\beta_{t\left[ i \right]}$ representing the treatment effect modification effects of covariables.

The interaction term for the reference treatment $\beta_{1}$ is constrained to zero so that the model is identified. The remaining interactions terms may be treatment-specific fixed effects or common across treatments. Therefore:

${\beta_{1}=0; \beta}_{2,\ldots,T}=\beta$ [5]

If common interaction terms are incorporated, then the results of the analysis will depend on which treatment is selected as the reference treatment. It may be helpful to compare the results from models using different treatments as the reference. Before selecting the common interaction term model it may be helpful to review estimates from the treatment-specific model.

## Random treatment effects model

In the arm-based parameterisation, a multivariate normal distribution is generated by including a study arm-specific random effect, $\delta_{s\left[ i \right], t[i]}$. Equation 5 is therefore extended to:

$\eta\left[ i \right]=\mu_{s\left[ i \right]}+d_{t\left[ i \right]}+\alpha.x\left[ i \right]+\beta_{t\left[ i \right]}.x\left[ i \right]+\delta_{s\left[ i \right],t\left[ i \right]}$ [6]

where:

$\delta_{s\left[ i \right],t\left[ i \right]}\sim N\left( 0,\frac{\sigma^{2}}{2} \right)$. [7]

## As in the contrast-based parameterisation, $\boldsymbol{\sigma}^{\boldsymbol{2}}$ represents the random effect variance. As this is assumed to be constant across all contrasts within the network, each individual treatment response is associated with a random effect variance of $\frac{\boldsymbol{\sigma}^{\boldsymbol{2}}}{\boldsymbol{2}}$ . This reflects the variation in response observed in individual arms.

## Correlation in random treatment effects in studies with 3 or more arms

The correlation in the random variation in treatment effects within multi-arm studies arises as treatment effect estimates within each study are jointly dependent on the variation in response in the base treatment arm of the study. The model in equations 6 and 7 produces the correct covariance for the common variance random effects model. For example, consider a single study, *s*, comparing treatments 1, 2, and 3. The random effect variance for the treatment effect contrast comparing treatments 1 and 2 is:

$Var\left( \delta_{s,2}- \delta_{s,1} \right)= \frac{\sigma^{2}}{2}+ \frac{\sigma^{2}}{2}= \sigma^{2}$ [8]

The covariance of the random effects for the contrasts comparing treatments 1 and 2 and treatments 1 and 3 for that study is:

$Cov\left( \delta_{s,2}- \delta_{s,1}, \delta_{s,3}- \delta_{s,1} \right)=Cov\left( \delta_{s,1}, \delta_{s,1} \right)= \frac{\sigma^{2}}{2}$ [9]

# Models explored in this analysis

The specifics of individual models fitted for this analysis using the framework described above are explained below. The following models were developed.

## Fixed treatment effects, no covariable adjustment

This model was achieved by removing terms related to covariables from equation 4:

$\eta\left[ i \right]=\mu_{s\left[ i \right]}+d_{t\left[ i \right]}$ [10]

## Fixed treatment effects, common covariable adjustment

This was achieved by combining equations 5 and 6:

$\eta\left[ i \right]=\mu_{s\left[ i \right]}+d_{t\left[ i \right]}+\alpha.x\left[ i \right]+\beta_{t\left[ i \right]}.x\left[ i \right]$ [11]

where:

${\beta_{1}=0; \beta}_{2,\ldots,T}=\beta$ [12]

## Fixed treatment effects, treatment-specific covariable adjustment

This is simply equation 11 without the constraint that $\beta_{t\left[ i \right]}$ coefficients are equal, ie:

$\eta\left[ i \right]=\mu_{s\left[ i \right]}+d_{t\left[ i \right]}+\alpha.x\left[ i \right]+\beta_{t\left[ i \right]}.x\left[ i \right]$ [13]

where:

$\beta_{1}=0;\beta_{2,\ldots,T}\sim N(0, 0.001)$ [14]

## Random treatment effects, no covariable adjustment

This is achieved by combining equations 6 and 7, and removing the covariable related terms, or equivalently adding random treatment effects to the equivalent fixed treatment effects model, ie:

$\eta\left[ i \right]=\mu_{s\left[ i \right]}+d_{t\left[ i \right]}+\delta_{s\left[ i \right],t\left[ i \right]}$ [15]

where:

$\delta_{s\left[ i \right],t\left[ i \right]}\sim N\left( 0,\frac{\sigma^{2}}{2} \right)$ [16]

## Random treatment effects, common covariable adjustment

This is achieved by adding covariable related terms to equation 15 and setting covariable related coefficients to be equal, ie:

$\eta\left[ i \right]=\mu_{s\left[ i \right]}+d_{t\left[ i \right]}+ \alpha.x\left[ i \right]+\beta_{t\left[ i \right]}.x\left[ i \right]+\delta_{s\left[ i \right],t\left[ i \right]}$ [17]

where:

$\delta_{s\left[ i \right],t\left[ i \right]}\sim N\left( 0,\frac{\sigma^{2}}{2} \right)$and ${\beta_{1}=0; \beta}_{2,\ldots,T}=\beta$ [18]

## Random treatment effects, treatment-specific covariable adjustment

This is achieved by relaxing the constraint that the covariable related coefficients are equal, ie:

$\eta\left[ i \right]=\mu_{s\left[ i \right]}+d_{t\left[ i \right]}+ \alpha.x\left[ i \right]+\beta_{t\left[ i \right]}.x\left[ i \right]+\delta_{s\left[ i \right],t\left[ i \right]}$ [19]

where instead:

$\delta_{s\left[ i \right],t\left[ i \right]}\sim N\left( 0,\frac{\sigma^{2}}{2} \right)$ and $\beta_{1}=0; \beta_{2,\ldots,T}\sim N(0, 0.001)$ [20]

# Results

Only the models providing some covariable adjustment were fitted. Results from the fitted models are shown in the main paper. The additional results presented in this additional file relate to the $\alpha$ and $\beta_{t}$ coefficients.

**Mean change in BCVA**

Model coefficients for NMA analyses for mean change in BCVA, a continuous outcome, are shown in Table A1. There was a positive association between baseline BCVA and mean change in BCVA across patients in studies in the NMA, as shown by the positive coefficient value for alpha. However, the beta coefficients were broadly negative, indicating that a greater positive change compared with the reference treatment was observed at lower baseline BCVA values.

**Odds of a 10-letter gain**

Model coefficients for NMA analyses for odds of a 10-letter gain, a binary outcome, are shown in Table A2. A higher BCVA at baseline was associated with a decreased chance of achieving a 10-letter response after 1 year, as shown by the generally negative alpha and beta coefficients.

**Odds of a 15-letter gain**

Model coefficients for NMA analyses for odds of a 15-letter gain, a binary outcome, are shown in Table A3. A higher BCVA at baseline was associated with a decreased chance of achieving a 15-letter response after 1 year, as shown by the negative coefficients.

Table A1 Mean (standard deviations) of NMA model coefficients relating to covariable adjustment: mean change in BCVA

| Network | Model | Model coefficients | | | | | | | |
| --- | --- | --- | --- | --- | --- | --- | --- | --- | --- |
|  |  | $\alpha$ | $\beta$ | $\beta_{1}$ | $\beta_{2}$ | $\beta_{3}$ | $\beta_{4}$ | $\beta_{5}$ | $\beta_{6}$ |
| Full | Fixed effect, common covariable adjustment | 0.8531 (0.0154) | -0.1572 (0.0196) | 0 |  |  |  |  |  |
|  | Random effects, common covariable adjustment | 0.8503 (0.0153) | -0.1431 (0.0202) | 0 |  |  |  |  |  |
| IPD only | Fixed effect, common covariable adjustment | 0.8578 (0.0152) | -0.1480 (0.0199) | 0 |  |  |  |  |  |
|  | Fixed effect, treatment-specific covariable adjustment | 0.8579 (0.0151) |  | 0 | -0.0846 (0.0262) | -0.2035 (0.0304) | -0.0877 (0.0374) | -0.0727 (0.0416) | -0.3225 (0.0417) |
|  | Random effects, common covariable adjustment | 0.8503 (0.0153) | -0.1392 (0.0201) | 0 |  |  |  |  |  |
|  | Random effects, treatment-specific covariable adjustment | 0.8503 (0.0151) |  | 0 | -0.0756 (0.0266) | -0.1933 (0.0302) | -0.0805 (0.0376) | -0.0648 (0.0415) | -0.315 (0.0421) |

**Table A2: Mean (standard deviations) of NMA model coefficients relating to covariable adjustment: log odds of 10-letter gain**

| Network | Model | Model coefficients | | | | | | | |
| --- | --- | --- | --- | --- | --- | --- | --- | --- | --- |
|  |  | $\alpha$ | $\beta$ | $\beta_{1}$ | $\beta_{2}$ | $\beta_{3}$ | $\beta_{4}$ | $\beta_{5}$ | $\beta_{6}$ |
| Full | Fixed effect, common covariable adjustment | -0.0322 (0.0070) | -0.0171 (0.0092) | 0 |  |  |  |  |  |
|  | Random effects, common covariable adjustment | -0.0346 (0.0070) | -0.0138 (0.0092) | 0 |  |  |  |  |  |
| IPD only | Fixed effect, common covariable adjustment | -0.0328 (0.0070) | -0.0156 (0.0092) | 0 |  |  |  |  |  |
|  | Fixed effect, treatment-specific covariable adjustment | -0.0327 (0.0069) |  | 0 | 0.0015 (0.0118) | -0.0173 (0.0140) | -0.0298 (0.0197) | -0.0228 (0.0186) | -0.0548 (0.0212) |
|  | Random effects, common covariable adjustment | -0.0327 (0.0070) | -0.0127 (0.0092) | 0 |  |  |  |  |  |
|  | Random effects, treatment-specific covariable adjustment | -0.0353 (0.0071) |  | 0 | 0.0040 (0.0118) | -0.0128 (0.0140) | -0.0274 (0.0200) | -0.0203 (0.0186) | -0.0524 (0.0216) |

| Network | Model | Model coefficients | | | | | | | |
| --- | --- | --- | --- | --- | --- | --- | --- | --- | --- |
|  |  | $\alpha$ | $\beta$ | $\beta_{1}$ | $\beta_{2}$ | $\beta_{3}$ | $\beta_{4}$ | $\beta_{5}$ | $\beta_{6}$ |
| Full | Fixed effect, common covariable adjustment | -0.0541 (0.0088) | -0.0066 (0.0108) | 0 |  |  |  |  |  |
|  | Random effects, common covariable adjustment | -0.0541 (0.0092) | -0.0067 (0.0111) | 0 |  |  |  |  |  |
| IPD only | Fixed effect, common covariable adjustment | -0.0527 (0.0090) | -0.0086 (0.0112) | 0 |  |  |  |  |  |
|  | Fixed effect, treatment-specific covariable adjustment | -0.0526 (0.0089) |  | 0 | -0.0021 (0.0132) | -0.0160 (0.0166) | -0.0069 (0.0215) | -0.0084 (0.0211) | -0.0267 (0.0216) |
|  | Random effects, common covariable adjustment | -0.0533 (0.0090) | -0.0079 (0.0111) | 0 |  |  |  |  |  |
|  | Random effects, treatment-specific covariable adjustment | -0.0534 (0.0091) |  | 0 | -0.0013 (0.0133) | -0.0153 (0.0167) | -0.0061 (0.0219) | -0.0076 (0.0212) | -0.0260 (0.0216) |

**Table A3: Mean (standard deviations) of NMA model coefficients in relation to covariable adjustment: log odds of 15-letter gain**

# References

1. Hawkins N, Scott DA, Woods B. ‘Arm-based’ parameterization for network meta-analysis. Res Syn Meth. 2016;7:306–13.

2. Achana FA, Cooper NJ, Dias S, et al. Extending methods for investigating the relationship between treatment effect and baseline risk from pairwise meta-analysis to network meta-analysis. Stat Med. 2013;32:752-771.
