# Supplementary material for: An efficacy comparison of anti-vascular growth factor agents and laser photocoagulation in diabetic macular edema: a network meta-analysis incorporating individual patient-level data
Source: BMC Ophthalmol. 2018 Dec 27;18:340. doi: 10.1186/s12886-018-1006-9 (PMC6307247; doi:10.1186/s12886-018-1006-9)
Supplement: Supplementary file 2 — Appendix 2. Treatment regimens classified and included in the NMA. (DOCX 64 kb) [file 12886_2018_1006_MOESM2_ESM.docx]

**Additional file 2**

**Appendix 2**

**Treatment regimens classified and included in the NMA**

| Study | Treatment arm | Dose | Regimen | Median injections over 12 months (n) | Classification of node in NMA |
| --- | --- | --- | --- | --- | --- |
| LUCIDATE [1] | Laser | – | Proactive then reactive  (fixed q4 then PRN) | – | Laser |
|  | Ranibizumab | 0.5 mg | Proactive then reactive  (fixed q4 then PRN) | – | Ranibizumab 0.5 mg PRN |
| Protocol I [2] | Laser + sham injections | – | Proactive then reactive  (fixed q4 then PRN) | – | Laser |
|  | Ranibizumab + prompt laser | 0.5 mg | Proactive then reactive  (fixed q4 then PRN) | 8 | Ranibizumab 0.5 mg + laser |
|  | Ranibizumab + deferred laser | 0.5 mg | Proactive then reactive  (fixed q4 then PRN) | 9 | Ranibizumab 0.5 mg PRN |
|  | IVTA + laser | 4 mg | Proactive then reactive  (fixed q4 then PRN) | 3 | IVTA 4 mg q4/PRN + laser |
| Protocol J [3] | IVTA + laser | 4 mg | Proactive (fixed) | – | IVTA 4 mg q4 + laser |
|  | Ranibizumab + laser | 0.5 mg | Proactive  (fixed) | – | Ranibizumab 0.5 mg PRN + laser |
|  | Laser | – | Proactive (fixed) | – | Laser |
| Protocol T [4] | IVT-AFL | 2 mg | Proactive  (fixed q4) | 9 | IVT-AFL 2q8 |
|  | Bevacizumab | 1.25 mg | Proactive  (fixed q4) | 10 | Bevacizumab 1.25 mg |
|  | Ranibizumab | 0.3 mg | Proactive  (fixed q4) | 10 | Ranibizumab 0.3 mg q4 |
| RESPOND [5] | Ranibizumab | 0.5 mg | Proactive then reactive  (fixed q4 then PRN) | – | Ranibizumab 0.5 mg PRN |
|  | Ranibizumab + laser | 0.5 mg | Proactive then reactive  (fixed q4 then PRN) | – | Ranibizumab 0.5 mg PRN + laser |
|  | Laser | – | Proactive then reactive  (fixed then PRN) | – | Laser |
| RESTORE [6] | Ranibizumab | 0.5 mg | Proactive then reactive  (fixed q4 then PRN) | 7 | Ranibizumab 0.5 mg PRN |
|  | Ranibizumab + laser | 0.5 mg | Proactive then reactive  (fixed q4 then PRN) | 7 | Ranibizumab 0.5 mg PRN + laser |
|  | Laser | – | Proactive then reactive  (fixed q4 then PRN) | – | Laser |
| RETAIN [7] | Ranibizumab | 0.5 mg | Proactive then reactive  (fixed q4 then PRN) | – | Ranibizumab 0.5 mg PRN |
|  | Ranibizumab | 0.5 mg | T&E | – | Ranibizumab 0.5 mg T&E |
|  | Ranibizumab + laser | – | T&E | – | Ranibizumab 0.5 mg T&E + laser |
| REVEAL [8] | Laser + sham injections | – | Proactive then reactive  (fixed q4 then PRN) | – | Laser |
|  | Ranibizumab + sham laser | 0.5 mg | Proactive then reactive  (fixed q4 then PRN) | 7.8 (mean) | Ranibizumab 0.5 mg PRN |
|  | Ranibizumab + prompt laser | 0.5 mg | Proactive then reactive  (fixed q4 then PRN) | 7.0 (mean) | Ranibizumab 0.5 mg PRN + laser |
| RIDE [9] | Laser + sham injections | – | Proactive  (fixed q4) | – | Laser |
|  | Ranibizumab + laser | 0.3 mg | Proactive  (fixed q4) | – | Ranibizumab 0.3 mg q4 + laser |
|  | Ranibizumab + laser | 0.5 mg | Proactive  (fixed q4) | – | Ranibizumab 0.5 mg q4 + laser |
| RISE [9] | Laser + sham injections | – | Proactive  (fixed q4) | – | Laser |
|  | Ranibizumab + deferred laser | 0.3 mg | Proactive  (fixed q4) | – | Ranibizumab 0.3 mg q4 + laser |
|  | Ranibizumab + laser | 0.5 mg | Proactive  (fixed q4) | – | Ranibizumab 0.5 mg q4 + laser |
| VISTA-DME [10] | Laser + sham injections | – | – | – | Laser |
|  | IVT-AFL + sham laser | 2 mg | Proactive  (fixed 2q4 then 2q8) | 9 | IVT-AFL 2q8 |
| VIVID-DME [10] | Laser + sham injections | – | – | – | Laser |
|  | IVT-AFL + sham laser | 2 mg | Proactive  (fixed 2q4 then 2q8) | 9 | IVT-AFL 2q8 |
| VIVID-EAST [11] | Laser | – | – | – | Laser |
|  | IVT-AFL | 2 mg | Proactive  (fixed 2q4 then 2q8) | – | IVT-AFL 2q8 |

q4, every 4 weeks; q8, every 8 weeks; 2q4, 2 mg every 4 weeks; 2q8, 2 mg every 8 weeks; IVTA, intravitreal triamcinolone acetonide; IVT-AFL, intravitreal aflibercept; NMA, network meta-analysis; PRN, as-needed; T&E, treat-and-extend.

**References**

1. Comyn O, Sivaprasad S, Peto T, et al. A randomized trial to assess functional and structural effects of ranibizumab versus laser in diabetic macular edema (the LUCIDATE study). Am J Ophthalmol. 2014;157:960-70.

2. Diabetic Retinopathy Clinical Research Network, Elman MJ, Aiello LP, et al. Randomized trial evaluating ranibizumab plus prompt or deferred laser or triamcinolone plus prompt laser for diabetic macular edema. Ophthalmology. 2010;117:1064-1077 e35.

3. Diabetic Retinopathy Clinical Research Network, Googe J, Brucker AJ, et al. Randomized trial evaluating short-term effects of intravitreal ranibizumab or triamcinolone acetonide on macular edema after focal/grid laser for diabetic macular edema in eyes also receiving panretinal photocoagulation. Retina. 2011;31:1009-27.

4. Diabetic Retinopathy Clinical Research Network, Wells JA, Glassman AR, et al. Aflibercept, bevacizumab, or ranibizumab for diabetic macular edema. N Engl J Med. 2015;372:1193-203.

5. Clinicaltrials.gov. Safety, efficacy and cost-efficacy of ranibizumab (monotherapy or combination with laser) in the treatment of diabetic macular edema (DME) (RESPOND). NCT01135914. <https://www.clinicaltrials.gov/ct2/show/NCT01135914?term=RESPOND&cond=DME&rank=1>. Accessed May 7, 2018.

6. Mitchell P, Bandello F, Schmidt-Erfurth U, et al. The RESTORE study: ranibizumab monotherapy or combined with laser versus laser monotherapy for diabetic macular edema. Ophthalmology. 2011;118:615-25.

7. Prunte C, Fajnkuchen F, Mahmood S, et al. Ranibizumab 0.5 mg treat-and-extend regimen for diabetic macular oedema: the RETAIN study. Br J Ophthalmol. 2016;100:787-95.

8. Ishibashi T, Li X, Koh A, et al. The REVEAL study: ranibizumab monotherapy or combined with laser versus laser monotherapy in Asian patients with diabetic macular edema. Ophthalmology. 2015;122:1402-15.

9. Nguyen QD, Brown DM, Marcus DM, et al. Ranibizumab for diabetic macular edema: results from 2 phase III randomized trials: RISE and RIDE. Ophthalmology. 2012;119:789-801.

10. Korobelnik JF, Do DV, Schmidt-Erfurth U, et al. Intravitreal aflibercept for diabetic macular edema. Ophthalmology. 2014;121:2247-54.

11. Clinicaltrials.gov. Efficacy and safety of VEGF Trap Eye in diabetic macular edema (DME) with central involvement (VIVID EAST). NCT01783886. <https://www.clinicaltrials.gov/ct2/show/NCT01783886?term=VIVID+EAST&rank=1>. Accessed May 7, 2018.
