# Supplementary material for: An efficacy comparison of anti-vascular growth factor agents and laser photocoagulation in diabetic macular edema: a network meta-analysis incorporating individual patient-level data
Source: BMC Ophthalmol. 2018 Dec 27;18:340. doi: 10.1186/s12886-018-1006-9 (PMC6307247; doi:10.1186/s12886-018-1006-9)
Supplement: Supplementary file 3 — Appendix 3. Overview of outcomes of studies included in the NMA. (DOCX 68 kb) [file 12886_2018_1006_MOESM3_ESM.docx]

**Additional file 3**

**Appendix 3**

**Overview of outcomes of studies included in the NMA**

| **Study** | **Treatment arm** | **N** | **Mean (SD) change in BCVA, ETDRS letters** | **Patients gaining  ≥10 letters, %** | **Patients gaining  ≥15 letters, %** | **Mean (SD) change in CRT, µm** |
| --- | --- | --- | --- | --- | --- | --- |
| LUCIDATE [1] | Laser | 11 | -0.9 (n/a) | n/a | n/a | n/a |
|  | Ranibizumab | 22 | +6.1 (n/a) | n/a | n/a | n/a |
| Protocol I^a^ [2] | Laser + sham injections | 293 | +3 (13) | 13 | 15 | -102 (151)^d^ |
|  | Ranibizumab + prompt laser | 187 | +9 (11) | 20 | 30 | -131 (129)^d^ |
|  | Ranibizumab + deferred laser | 188 | +9 (12) | 19 | 28 | -137 (136)^d^ |
|  | IVTA + laser | 186 | +4 (13) | 12 | 21 | -127 (140)^d^ |
| Protocol J^b^ [3] | IVTA + laser | 109 | -5 (16) | 5 | 8 | -92 (115)^d^ |
|  | Ranibizumab + laser | 113 | -4 (21) | 11 | 13 | -39 (127)^d^ |
|  | Laser | 123 | -6 (17) | 8 | 5 | -5 (113)^d^ |
| Protocol T^a^ [4] | IVT-AFL | 224 | +13.3 (n/a) | Baseline letter score <69: 77  Baseline letter score 69-78: 50 | Baseline letter score <69: 67  Baseline letter score 69-78: 18 | -169 (138)^d^ |
|  | Bevacizumab | 218 | +9.7 (n/a) | Baseline letter score <69: 60  Baseline letter score 69-78: 45 | Baseline letter score <69: 41  Baseline letter score 69-78: 16 | -101 (121)^d^ |
|  | Ranibizumab | 218 | +11.2 (n/a) | Baseline letter score <69: 69  Baseline letter score 69-78: 50 | Baseline letter score <69: 50  Baseline letter score 69-78: 15 | -147 (134)^d^ |
| RESPOND^a^ [5] | Ranibizumab | 75 | +8.9 (7.8) | 52.1 | 21.1 | -143.5 (144.0) |
|  | Ranibizumab + laser | 73 | +8.2 (9.2) | 34.3 | 24.3 | -152.2 (139.3) |
|  | Laser | 72 | +0.3 (12.5) | 16.1 | 6.5 | -107.1 (143.9) |
| RESTORE^a^ [6] | Ranibizumab | 115 | +6.1 (6.4) | 37.4 | 22.6 | -118.7 (115.1) |
|  | Ranibizumab + laser | 118 | +5.9 (7.9) | 43.2 | 22.9 | -128.3 (114.3) |
|  | Laser | 111 | +0.8 (8.6) | 15.5 | 8.2 | -61.3 (132.3) |
| RETAIN^a^ [7] | Ranibizumab T&E | 128 | +6.1 (5.7) | n/a | n/a | 24.4^e^ |
|  | Ranibizumab PRN | 123 | +5.9 (5.5) | n/a | n/a | 23.2^e^ |
|  | Ranibizumab T&E + laser | 121 | +6.2 (6.0) | n/a | n/a | 27.1^e^ |
| REVEAL^a^ [8] | Laser + sham injections | 131 | +1.4 (6.5) | 13.3 | 7.8 | -58.6 (n/a)^f^ |
|  | Ranibizumab + sham laser | 133 | +5.9 (6.2) | 33.8 | 18.8 | -132.5 (n/a)^f^ |
|  | Ranibizumab + prompt laser | 132 | +5.7 (7.2) | 37.2 | 17.8 | -164.5 (n/a)^f^ |
| RIDE^c^ [9] | Laser + sham injections | 130 | +2.3 (n/a) | n/a | 12.3 | -125.8 (n/a)^g^ |
|  | Ranibizumab 0.3 mg + laser | 125 | +10.9 (n/a) | n/a | 33.6 | -259.8 (n/a)^g^ |
|  | Ranibizumab 0.5 mg + laser | 127 | +12.0 (n/a) | n/a | -45.7 | -270.7 (n/a)^g^ |
| RISE^c^ [9] | Laser + sham injections | 127 | +2.6 (n/a) | n/a | 18.1 | -133.4 (n/a)^g^ |
|  | Ranibizumab 0.3 mg + deferred laser | 125 | +12.5 (n/a) | n/a | 44.8 | -250.6 (n/a)^g^ |
|  | Ranibizumab 0.5 mg + laser | 125 | +11.9 (n/a) | n/a | 39.2 | -253.1 (n/a)^g^ |
| VISTA-DME^a^ [10] | Laser + sham injections | 154 | +0.2 (12.4) | 19.5 | 7.8 | -73.3 (196.7)^d^ |
|  | IVT-AFL 2q4 + sham laser | 154 | +12.5 (9.5) | 64.9 | 41.6 | -185.9 (150.7)^d^ |
|  | IVT-AFL 2q8 + sham laser | 151 | +10.7 (8.2) | 58.3 | 31.1 | -183.1 (153.5)^d^ |
| VIVID-DME^a^ [10] | Laser + sham injections | 132 | +10.5 (9.5) | 25.8 | 9.1 | -66.2 (139.0)^d^ |
|  | IVT-AFL 2q4 + sham laser | 136 | +10.7 (9.3) | 54.4 | 32.4 | -195.0 (146.6)^d^ |
|  | IVT-AFL 2q8 + sham laser | 135 | +1.2 (10.6) | 53.3 | 33.3 | -192.4 (149.9)^d^ |
| VIVID-EAST^a^ [11] | Laser | 124 | -0.2 (13.5) | 23.4 | 12.1 | -109.3 (206.9) |
|  | IVT-AFL 2q4 + sham laser | 127 | +13.7 (8.4) | 70.9 | 43.3 | -238.7 (172.5) |
|  | IVT-AFL 2q8 + sham laser | 127 | +12.8 (9.5) | 62.7 | 36.5 | -234.7 (160.8) |

^a^1-year results; ^b^56-week results; ^c^2-year results; ^d^CST; ^e^% change CSFT; ^f^CRST, ^g^CFT

2q4, 2 mg IVT-AFL every 4 weeks; 2q8, 2 mg IVT-AFL every 8 weeks after 5 initial monthly doses; CFT, central foveal thickness; CRT, central retinal thickness; CRST, central retinal subfield thickness; CST, central subfield thickness; n/a, not applicable; PRN, pro re nata; SD, standard deviation; T&E, treat-and-extend

**References**

1. Comyn O, Sivaprasad S, Peto T, et al. A randomized trial to assess functional and structural effects of ranibizumab versus laser in diabetic macular edema (the LUCIDATE study). Am J Ophthalmol. 2014;157:960-70.

2. Diabetic Retinopathy Clinical Research Network, Elman MJ, Aiello LP, et al. Randomized trial evaluating ranibizumab plus prompt or deferred laser or triamcinolone plus prompt laser for diabetic macular edema. Ophthalmology. 2010;117:1064-1077 e35.

3. Diabetic Retinopathy Clinical Research Network, Googe J, Brucker AJ, et al. Randomized trial evaluating short-term effects of intravitreal ranibizumab or triamcinolone acetonide on macular edema after focal/grid laser for diabetic macular edema in eyes also receiving panretinal photocoagulation. Retina. 2011;31:1009-27.

4. Diabetic Retinopathy Clinical Research Network, Wells JA, Glassman AR, et al. Aflibercept, bevacizumab, or ranibizumab for diabetic macular edema. N Engl J Med. 2015;372:1193-203.

5. Clinicaltrials.gov. Safety, efficacy and cost-efficacy of ranibizumab (monotherapy or combination with laser) in the treatment of diabetic macular edema (DME) (RESPOND). NCT01135914. . <https://www.clinicaltrials.gov/ct2/show/NCT01135914?term=RESPOND&cond=DME&rank=1>. Accessed May 7, 2018.

6. Mitchell P, Bandello F, Schmidt-Erfurth U, et al. The RESTORE study: ranibizumab monotherapy or combined with laser versus laser monotherapy for diabetic macular edema. Ophthalmology. 2011;118:615-25.

7. Prunte C, Fajnkuchen F, Mahmood S, et al. Ranibizumab 0.5 mg treat-and-extend regimen for diabetic macular oedema: the RETAIN study. Br J Ophthalmol. 2016;100:787-95.

8. Ishibashi T, Li X, Koh A, et al. The REVEAL study: ranibizumab monotherapy or combined with laser versus laser monotherapy in Asian patients with diabetic macular edema. Ophthalmology. 2015;122:1402-15.

9. Nguyen QD, Brown DM, Marcus DM, et al. Ranibizumab for diabetic macular edema: results from 2 phase III randomized trials: RISE and RIDE. Ophthalmology. 2012;119:789-801.

10. Korobelnik JF, Do DV, Schmidt-Erfurth U, et al. Intravitreal aflibercept for diabetic macular edema. Ophthalmology. 2014;121:2247-54.

11. Clinicaltrials.gov. Efficacy and safety of VEGF Trap Eye in diabetic macular edema (DME) with central involvement (VIVID EAST). NCT01783886. . <https://www.clinicaltrials.gov/ct2/show/NCT01783886?term=VIVID+EAST&rank=1>. Accessed May 7, 2018.
